# Sleep Quality and Mental Health Among Medical Students: A Cross-Sectional Study

**DOI:** 10.3390/jcm14072274

**Published:** 2025-03-26

**Authors:** Stipe Vidović, Nada Rakić, Stela Kraštek, Ana Pešikan, Dunja Degmečić, Lada Zibar, Irena Labak, Marija Heffer, Zenon Pogorelić

**Affiliations:** 1 Faculty of Medicine, University Josip Juraj Strossmayer, 31 000 Osijek, Croatia; 2 National Memorial Hospital Vukovar, 32 000 Vukovar, Croatia; 3 School of Medicine Split, University of Split, 21 000 Split, Croatia; 4 Department of Psychiatry and Psychological Medicine, Faculty of Medicine, University Josip Juraj Strossmayer, 31 000 Osijek, Croatia; 5 Department of Biology, University Josip Juraj Strossmayer, 31 000 Osijek, Croatia; 6 Department of Surgery, School of Medicine, University of Split, 21 000 Split, Croatia; 7 Department of Pediatric Surgery, University Hospital of Split, 21 000 Split, Croatia

**Keywords:** sleep quality, sleep disorders, depression, anxiety, stress, mental health, mental disorders, student, medical student

## Abstract

**Background:** Sleep disturbances and mental health disorders represent a significant public health concern. Medical students, in particular, experience intense academic pressure, long study hours, irregular schedules, and the emotional burden of clinical training, all of which may contribute to the development of sleep disturbances and mental health issues. This study aims to assess sleep quality and the prevalence of symptoms of depression, anxiety, and stress among medical students, as well as their interrelationships. **Methods:** This multicentric cross-sectional study was conducted in January and February 2025 among medical students from two medical faculties in Croatia. Sleep quality was assessed using the Pittsburgh Sleep Quality Index (PSQI), while symptoms of depression, anxiety, and stress were evaluated using Depression, Anxiety, and Stress Scale-21 (DASS-21). **Results:** The study included 386 participants, of whom 96 were male and 290 were female students. It was found that 67.9% of students had poor sleep quality, while symptoms of depression, anxiety, and stress were reported by 38.8%, 45.3%, and 40.4% of participants, respectively. Female students exhibited higher PSQI scores (U = 10,205, *p* < 0.001), as well as higher levels of depression (U = 10,372, *p* < 0.001), anxiety (U = 10,328, *p* < 0.001), and stress scores (U = 10,560, *p* < 0.001). Additionally, significant moderate positive correlations were observed between the total PSQI score and depression (ρ = 0.566, *p* < 0.001), anxiety (ρ = 0.489, *p* < 0.001), and stress scores (ρ = 0.503, *p* < 0.001). Moreover, an increase in depression (β = 0.178, *p* < 0.001) and anxiety scores (β = 0.141, *p* < 0.001) contributed to a higher total PSQI score, indicating poorer sleep quality. **Conclusions:** A high prevalence of poor sleep quality and symptoms of depression, anxiety, and stress was observed among medical students, with female students exhibiting higher levels of these variables. Furthermore, poorer sleep quality was associated with higher levels of depression, anxiety, and stress.

## 1. Introduction

Upon entering higher education, students typically undergo significant changes in their daily routines and lifestyles while simultaneously facing challenges such as managing increasing academic demands, making career decisions, and relocating for educational or professional opportunities [1,2,3]. In addition to academic stress, they often encounter financial concerns and social pressure [4]. Moreover, many students struggle to maintain a balanced diet, engage in regular physical activity, and establish consistent sleep patterns due to fluctuating workloads and social commitments [5,6,7]. Medical students, in particular, face unique challenges due to the highly demanding and rigorous nature of their education [8,9]. They must absorb vast amounts of complex medical knowledge within a short period, often resulting in intense study sessions, prolonged academic engagement, and irregular sleep patterns [8,10]. Furthermore, they undergo clinical rotations, where they balance theoretical learning with hands-on work with patients, endure long shifts, and navigate emotionally distressing situations, including exposure to suffering and death [11,12]. These factors may contribute to the high prevalence of poor sleep quality and mental health disorders among medical students [13,14].

A systematic review and meta-analysis by Binjabr et al. (2023) examined the sleep quality of 59,427 medical students from 31 countries worldwide between 2000 and 2023 [15]. The study found that 55.6% (95% confidence interval (CI), 51.5–59.7) of students self-reported poor sleep quality according to the Pittsburgh Sleep Quality Index (PSQI). Furthermore, Jahrami et al. (2020), in their systematic review and meta-analysis of 18,619 students from 13 countries worldwide between 2000 and 2018, observed a similar prevalence of poor sleep quality (55% [95% CI, 48.0–62.0]) among medical students according to the PSQI [16].

Furthermore, a systematic review and meta-analysis by Daniali et al. (2023) indicated that, compared to the general population, students worldwide exhibit higher levels of depression, anxiety, and stress [17]. A meta-analysis and systematic review by Wang et al. (2023) found that, on a global scale, 37% (95% CI, 32–42) of students experienced symptoms of depression, 29% (95% CI, 19–25) experienced anxiety, and 23% (95% CI, 8–39) experienced stress [18]. A similar prevalence of anxiety symptoms (33.8% [95% CI, 29.2–38.7]) among medical students worldwide was reported by Quek et al. (2019) [19]. Moreover, a meta-analysis and systematic review by Lin et al. (2024), which examined 132,068 medical students worldwide, found that 48% (95% CI, 43–52) experienced symptoms of depression, while 45% (95% CI, 40–49) experienced anxiety [20]. Some studies suggest that medical students exhibit higher levels of depression, anxiety, and stress compared to non-medical students [21,22], while others have not observed a significant difference in the prevalence of these conditions [23,24,25,26,27,28,29].

This study aims, for the first time in Croatia, to simultaneously assess sleep quality and levels of depression, anxiety, and stress among medical students, as well as to evaluate the interconnections between these variables.

## 2. Methods

### 2.1. Study Design

The study was designed as a multicentric cross-sectional study among medical students from the Faculty of Medicine Osijek, University Josip Juraj Strossmayer in Osijek, Croatia, and the University of Split, School of Medicine, Split, Croatia.

### 2.2. Participants and Recruitment

Participants were invited via their official school email addresses to complete the questionnaire hosted on Google Forms. An email was sent to the participants containing a document with information about the study’s structure, objectives, methodology, and the role of participants in case they decided to take part. The importance of providing honest responses was emphasized. Participation was voluntary and anonymous, and participants could withdraw from completing the questionnaire at any time. At the end of the document, a link to the questionnaire was provided. Before starting the survey, participants were required to confirm their consent to participate in the study. The first invitation to participate was sent via email on 25 January 2025, followed by a reminder on 5 February 2025. The survey was closed on 10 February 2025.

### 2.3. Survey

The survey consisted of three parts and included 63 questions, with an estimated completion time of 10–15 min. The first part gathered information on the participants’ sociodemographic characteristics, including age, sex, year of study, self-reported grade point average (GPA), place of residence, relationship status, self-reported material/financial status, and the use of cigarettes and alcohol.

The second part employed the Croatian version of the Pittsburgh Sleep Quality Index (PSQI) to evaluate the sleep quality of participants over the previous month [30,31]. The PSQI consists of 19 questions distributed across seven components: subjective sleep quality, sleep latency, sleep duration, sleep efficiency, sleep disturbances, use of sleeping medication, and daytime dysfunction. Each component is scored on a scale from 0 to 3. The total PSQI score is calculated by summing the scores from all seven components, resulting in a cumulative score ranging from 0 to 21, where a higher score indicates poorer sleep quality. Participants with a total PSQI score of 5 or less are classified as good sleepers, whereas those scoring above 5 are classified as poor sleepers [31].

The third part of the questionnaire assessed the presence of symptoms of depression, anxiety, and stress using the Croatian version of the Depression Anxiety Stress Scale 21 (DASS-21) [32,33]. DASS-21 is used to identify and differentiate symptoms of three emotional states: depression, anxiety, and stress. The DASS-21 scale is divided into three subscales: Depression subscale (DASS-21 D), Anxiety subscale (DASS-21 A), and Stress subscale (DASS-21 S). Each subscale contains seven questions, with respondents selecting one of four possible responses on a Likert scale from 0 to 3. Zero (0) indicates “Did not apply to me at all”, 1 indicates “Applied to me to some degree, or some of the time”, 2 indicates “Applied to me to a considerable degree, or a good part of the time”, and 3 indicates “Applied to me very much, or most of the time”. The total scores for the DASS-21 D, DASS-21 A, and DASS-21 S subscales are calculated by summing the responses. Based on these total subscale scores, participants were categorized into one of five levels of symptoms: normal, mild, moderate, severe, and extremely severe for depression, anxiety, and stress [33].

### 2.4. Sample Size Calculation

The sample size was calculated based on the total of 1015 medical students from the Faculty of Medicine Osijek (*n* = 459) and the School of Medicine Split (*n* = 556). Given a population size of 1015, a margin of error of 5%, a confidence level of 95%, and a response distribution of 50%, the minimum required sample size was 279.

### 2.5. Statistical Methods

The normality of the data and the homogeneity of variances were verified using the Kolmogorov–Smirnov test and Levene’s test, respectively. Nominal variables were presented as absolute and relative frequencies (percentages), while numerical variables were presented using the median and interquartile range (IQR). The Mann–Whitney U test was used to compare the total PSQI score, depression, anxiety, and stress scores by sex. Spearman’s rank correlation coefficients were used to assess the relationships between the PSQI component scores (subjective sleep quality, sleep latency, sleep duration, sleep efficiency, use of sleep medication, sleep disturbance, daytime dysfunction, and total PSQI) and the depression, anxiety, and stress scores. Multiple linear regression analysis was performed with depression, anxiety, and stress scores as independent variables and the total PSQI score as the dependent variable. The variance inflation factor (VIF) was used to assess multicollinearity among the independent variables. The level of statistical significance was set at *p* < 0.05.

## 3. Results

A total of 1015 students were invited to participate, and 410 respondents completed the survey, resulting in an overall response rate of 40.4% (Faculty of Medicine Osijek: 43.6%, School of Medicine Split: 37.8%). The completion rate was 97%, meaning that 24 respondents did not fully complete the survey and were excluded from further data analysis. Ultimately, 386 students fully completed the survey. The final sample exceeded the minimum required sample size calculated in the power analysis, indicating that the study maintained adequate statistical power. Of the total participants, 197 (51%) were from the Faculty of Medicine Osijek, and 189 (49%) were from the School of Medicine Split. Among them, 290 (75.1%) were female and 96 (24.9%) were male. The median age of participants was 21 (IQR: 19–23) years. Other sociodemographic characteristics are presented in Table 1.

It was found that 67.9% of participants had poor sleep quality (total PSQI score > 5). Furthermore, symptoms of depression, anxiety, and stress were present in 38.3%, 45.3%, and 40.4% of participants, respectively. Notably, severe and extremely severe levels of depression, anxiety, and stress were observed in 15.3%, 20.5%, and 16.6% of participants, respectively (Table 2).

In a sex-specific comparison, women had significantly higher total PSQI scores (U = 10,205, *p* < 0.001), subjective sleep quality scores (U = 10,447, *p* < 0.001), and daytime dysfunction scores (U = 10,922, *p* < 0.001). Furthermore, women had significantly higher depression (U = 10,372, *p* < 0.001), anxiety (U = 10,328, *p* < 0.001), and stress scores (U = 10,560, *p* < 0.001) compared to men (Table 3).

Significant moderate positive correlations were observed between the total PSQI score and depression (ρ = 0.566, *p* < 0.001), anxiety (ρ = 0.489, *p* < 0.001), and stress (ρ = 0.503, *p* < 0.001). The remaining correlations between PSQI subcategory scores and depression, anxiety, and stress are presented in Table 4.

Furthermore, a multiple linear regression analysis was conducted with depression, anxiety, and stress scores as independent variables and the total PSQI score as the dependent variable. The results indicated that both depression and anxiety were statistically significant predictors of the total PSQI score. Specifically, increases in depression (β = 0.178, t = 3.987, SE (standard error) = 0.045, *p* < 0.001) and anxiety scores (β = 0.141, t = 2.386, SE = 0.059, *p* < 0.001) were associated with increases in the total PSQI score, suggesting poorer sleep quality (Table 5). The variance inflation factors (VIF) for depression, anxiety, and stress scores were 2.573, 3.127, and 3.664, respectively, indicating that there is not a concerning level of multicollinearity among the independent variables.

## 4. Discussion

The results indicate a high prevalence of self-reported poor sleep quality, as well as depression, anxiety, and stress among medical students, with female students exhibiting poorer sleep quality and higher levels of these negative emotions. Furthermore, higher levels of depression, anxiety, and stress were found to be associated with poorer sleep quality among students.

Globally, a systematic review and meta-analysis by Binjabr et al. (2023), which included 109 studies and 59,427 students worldwide, found that 55.6% of students had poor sleep quality [15]. Another systematic review and meta-analysis conducted by Leow et al. (2023), covering 9466 medical students globally, reported that 57% of students had poor sleep quality [34]. These meta-analysis results suggest that the prevalence of poor sleep quality in our study was slightly higher based on the PSQI. Furthermore, sleep quality among students in Croatia was previously investigated by Štefan et al. (2018), who found that 37.6% of students experienced poor sleep quality [35]. Our results indicate a higher prevalence of poor sleep quality, which may be attributed to the fact that our participants were medical students whose study program is characterized by demanding academic schedules, extended study hours, and clinical rotations that disrupt regular sleep patterns [10,11,12]. Furthermore, elevated levels of stress, anxiety, irregular schedules, night shifts, and exposure to emotionally challenging situations may further contribute to sleep difficulties and poor sleep quality among students [10,11,12,13,14,15].

In this study, we identified a high prevalence of depression, anxiety, and stress among medical students. In Croatia, Romić et al. (2021) examined depression and anxiety levels among medical students at the University of Zagreb using the nine-item Patient Health Questionnaire (PHQ-9) and the Generalized Anxiety Disorder seven-item scale (GAD-7), reporting higher rates of depression (62.5%) and anxiety (75.3%) compared to our findings [36]. This discrepancy could be attributed to the fact that their study was conducted during the COVID-19 pandemic, a period marked by social isolation, fear of infection, feelings of loneliness and uncertainty, and financial concerns [37,38]. On a global scale, a systematic review and meta-analysis by Wang et al. (2023), which included 28 studies with a total of 436,799 students, reported slightly lower prevalence rates of depression (37% [95% CI, 32–42]), anxiety (29% [95% CI, 19–25]), and stress (23% [95% CI, 8–39]) compared to our results [18]. The high levels of depression, anxiety, and stress observed in students may stem from a combination of academic, financial, and social factors. The demanding nature of higher education, characterized by heavy workloads, frequent assessments, and high expectations, significantly contributes to psychological distress [39]. Additionally, financial strain, including tuition fees and living expenses, can further exacerbate anxiety and stress [4]. Social factors, such as adjusting to a new environment, maintaining interpersonal relationships, and potential feelings of isolation, may also play a role in the high prevalence of negative emotions [40,41]. Furthermore, inadequate support systems within educational institutions and limited access to mental health services may leave many students without the necessary psychological support, allowing mental health symptoms to worsen over time [42]. In this study, we found that 15.8% of students had sought professional psychological or psychiatric help, while as many as 42.5% were considering seeking such support. Given these findings, emphasis should be placed on improving the accessibility and availability of professional psychological services, as well as reducing the stigma associated with seeking mental health support [43].

Some studies suggest that female students report poorer sleep quality [44,45,46], while others have not observed a significant difference [47,48]. In our study, when comparing self-reported sleep quality based on total PSQI scores, female students exhibited poorer sleep quality than male students. Furthermore, when comparing negative emotions by sex, female students were found to report higher levels of depression, anxiety, and stress. This difference has also been observed in a systematic review and meta-analysis by Daniali et al. (2023), which confirmed that this pattern is consistent globally, with female students displaying higher levels of these negative emotions [17]. Several factors may contribute to this disparity. Psychologically, women tend to internalize emotions more frequently, which can intensify feelings of depression, anxiety, and stress [49]. Additionally, greater hormonal fluctuations in women may contribute to heightened mood variability and emotional responses [50]. Furthermore, societal and cultural expectations can impose unique pressures on young women, including the need to excel academically while also managing social and familial responsibilities [51]. These psychological and psychosocial stressors, along with hormonal factors, may not only contribute to elevated levels of negative emotions but also help explain the poorer sleep quality reported by female students [52]. Increased stress and emotional arousal are well-documented to negatively impact both sleep onset and maintenance, potentially resulting in more frequent sleep disturbances and overall reduced sleep quality among women [53].

Study results suggest that depression and anxiety were predictors of total PSQI scores, with higher levels of these negative emotions potentially contributing to poorer sleep quality among medical students. Furthermore, higher levels of depression, anxiety, and stress were found to be associated with poorer subjective sleep quality, longer sleep latency, shorter sleep duration, more pronounced sleep disturbances, and greater daytime dysfunction. These findings are consistent with previous research indicating that depression, anxiety, and stress can contribute to poorer sleep quality and increase the likelihood of developing sleep disorders [54,55]. The observed link may be explained by the impact of negative affective states on the circadian rhythm, which can lead to altered sleep patterns through mechanisms such as hormonal dysregulation [56]. For instance, cortisol levels, which become dysregulated in the chronic presence of negative emotions, may interfere with the suprachiasmatic nucleus, resulting in circadian disruption, delayed sleep onset, and altered sleep architecture [54,56]. Additionally, the prolonged persistence of negative emotional states can lead to epigenetic modifications that alter the expression of crucial circadian rhythm genes, such as CLOCK, BMAL1, PER, and CRY [55]. Disruptions in the expression or function of these genes may contribute to circadian misalignment, manifesting as sleep disorders and irregular sleep patterns [55,56]. It is noteworthy that studies suggest a bidirectional relationship between depression, anxiety, stress, and sleep quality. However, given that this is a cross-sectional study, it is not possible to draw conclusions regarding causality or the specific direction of this relationship [57].

This study had certain limitations. Given the cross-sectional nature of the study, causal relationships between sleep quality and symptoms of depression, anxiety, and stress cannot be established. Furthermore, to assess sleep quality in this study, we used the self-reported PSQI questionnaire, which relies on self-assessment and can lead to bias and data unreliability, as individuals may overestimate or underestimate their sleep problems. Further studies might benefit from incorporating more objective methods such as polysomnography, actigraphy, or qualitative methods using semi-structured interviews and sleep diaries to capture insights that may have been missed due to the self-reported measures used in the present study [58,59]. Furthermore, the DASS-21 questionnaire, which was utilized for assessing symptoms of depression, anxiety, and stress, is suitable for initial assessments but is not adequate for establishing definitive diagnoses of mental disorders. Applying thresholds for mild forms of depression, anxiety, and stress could potentially lead to inflated prevalence rates. Furthermore, although the DASS-21 is a validated and reliable self-report instrument for assessing symptoms of depression, anxiety, and stress, it includes items (e.g., “I couldn’t seem to experience any positive feeling at all”) that may potentially elicit or intensify negative emotions in some participants. This study did not—but should have—organized and provided contact information for professional psychological or psychiatric support that would be available to participants in case completing the questionnaire induced discomfort or emotional distress. Additionally, the study primarily focused on students’ mental health as a potential factor associated with sleep quality. Implementing a broader set of instruments would provide a more comprehensive understanding of the potential underlying causes of the high prevalence of poor sleep quality among students. In future studies, it would be useful to assess other variables such as physical activity, diet, use of blue light, and consumption of coffee and other caffeinated products. Additionally, the participants were students from the Faculty of Medicine in Osijek and the University of Split School of Medicine. Given that Croatia has three additional medical faculties—the Faculty of Medicine in Zagreb, the Faculty of Medicine in Rijeka, and the School of Medicine at the Catholic University of Croatia—for greater representativeness, it would be advisable to include medical students from all faculties in the country in future research.

## 5. Conclusions

A high prevalence of poor sleep quality and symptoms of depression, anxiety, and stress was observed among medical students, underscoring the need for further large-scale longitudinal studies to identify underlying risk factors. Furthermore, these findings emphasize the importance of developing effective strategies for the early detection of sleep and mental health disturbances, as well as the implementation of targeted interventions (e.g., stress management workshops, sleep hygiene education, time management training, mindfulness programs, and improved access to psychological support services) to enhance sleep quality and mental health in the student population.

## Figures and Tables

**Table 1:** Sociodemographic characteristics of participants (*n* = 386).

**Variables**		***n* (%) or Median (IQR)**
Sex	Male	96 (24.9)
	Female	290 (75.1)
Age (years)		21 (19–23)
BMI (kg/m^2^)	<18.5	21 (5.4)
	18.5–24.9	289 (74.9)
	25.0–29.9	64 (16.6)
	30.0–34.9	9 (2.3)
	35.0–39.9	1 (0.3)
	>40	2 (0.5)
Year of study	First	106 (27.5)
	Second	83 (21.5)
	Third	31 (8)
	Fourth	53 (13.7)
	Fifth	72 (18.7)
	Sixth	41 (10.6)
GPA		4.4 (4.0–4.8)
Place of residence	Urban	312 (80.8)
	Rural	74 (19.2)
Living	Alone	74 (19.2)
	With roommate	114 (29.5)
	With family	198 (51.3)
Material/financial status	Far below average	9 (2.3)
	Below average	34 (8.8)
	Average	197 (51)
	Above average	130 (33.7)
	Far above average	16 (4.1)
Relationship status	Single	146 (37.8)
	In relationship	240 (62.2)
Smoking	No	332 (86)
	Yes	54 (14)
Alcohol consumption	Never	71 (18.4)
	Sometimes	259 (67.1)
	1× a week	39 (10.1)
	2× a week	12 (3.1)
	≥3× a week	5 (1.3)
I am considering seeking professional psychological/psychiatric help	Yes	164 (42.5)
	No	222 (57.5)
I sought professional psychological/psychiatric help	Yes	61 (15.8)
	No	325 (84.2)

IQR, interquartile range; BMI, Body mass index; GPA, Grade point average.

**Table 2:** Participants self-reported sleep quality and mental health (*n* = 386).

Variables		*n* (%) or Median (IQR)
Sleep quality	Poor sleepers	262 (67.9)
	Good sleepers	124 (32.1)
PSQI subcategories scores	Subjective sleep quality	2 (1–2)
	Sleep latency	1 (1–2)
	Sleep duration	1 (1–2)
	Sleep efficiency	0 (0–0)
	Sleep disturbance	1 (1–1)
	Use of sleep medication	0 (0–0)
	Daytime dysfunction	1 (1–2)
	Total PSQI score	7 (5–9)
Depression category	Normal	238 (61.7)
	Mild	37 (9.6)
	Moderate	52 (13.5)
	Severe	29 (7.5)
	Extremely severe	30 (7.8)
Anxiety category	Normal	209 (54.1)
	Mild	55 (14.2)
	Moderate	43 (11.1)
	Severe	34 (8.8)
	Extremely severe	45 (11.7)
Stress category	Normal	230 (59.6)
	Mild	49 (12.7)
	Moderate	43 (11.1)
	Severe	47 (12.2)
	Extremely severe	17 (4.4)
DASS-21 scores	Depression score	3 (1–7)
	Anxiety score	3 (1–6.75)
	Stress score	6 (2–10)

IQR, interquartile range; PSQI, Pittsburgh Sleep Quality Index; DASS-21, Depression Anxiety Stress Scale 21.

**Table 3:** Sex-specific comparison of the total PSQI components, depression, anxiety, and stress scores (*n* = 386).

	Male (*n* = 96)		Female (*n* = 290)			
Variables	Median	IQR	Median	IQR	U	*p* *
Subjective sleep quality	1	(0–2)	2	(1–2)	10,447	<0.001
Sleep latency	1	(0–2)	1	(1–2)	12,875	0.25
Sleep duration	1	(0–2)	1	(1–2)	12,521	0.12
Sleep efficiency	0	(0–0)	0	(0–0)	12,998	0.151
Sleep disturbance	1	(1–1)	1	(1–2)	12,250	0.023
Use of sleep medication	0	(0–0)	0	(0–0)	13,792	0.786
Daytime dysfunction	1	(1–2)	1	(1–2)	10,922	<0.001
Total PSQI score	6	(4–8)	7	(2–9)	10,205	<0.001
Depression score	2	(1–5)	3	(2–8)	10,372	<0.001
Anxiety score	2	(0–4)	3	(1–7)	10,328	<0.001
Stress score	4	(1–8)	6.5	(3–11)	10,560	<0.001

IQR, interquartile range; PSQI, Pittsburgh Sleep Quality Index, * Mann–Whitney U test.

**Table 4:** Spearman’s rank correlation coefficients (ρ) between the PSQI subcategories, depression, anxiety, and stress scores (*n* = 386).

Variables	(1)	(2)	(3)	(4)	(4)	(5)	(6)	(7)	(8)	(9)	(10)
(1) Subjective sleep quality		0.169 ***	0.273 ***	0.069	0.312 ***	0.141 **	0.761 ***	0.739 ***	0.582 ***	0.459 ***	0.478 ***
(2) Sleep latency			0.091	0.158 **	0.412 ***	0.212 ***	0.109 *	0.555 ***	0.208 ***	0.193 ***	0.175 ***
(3) Sleep duration				0.228 ***	0.178 ***	0.093	0.234 ***	0.577 ***	0.24 ***	0.222 ***	0.262 ***
(4) Sleep efficiency					0.105 *	0.036	0.087	0.357 ***	0.101 *	0.05	0.088
(4) Sleep disturbance						0.238 ***	0.246 ***	0.560 ***	0.38 ***	0.432 ***	0.378 ***
(5) Use of sleep medication							0.081	0.338 ***	0.183 ***	0.152 **	0.161 **
(6) Daytime dysfunction								0.675 ***	0.493 ***	0.399 ***	0.413 ***
(7) Total PSQI score									0.566 ***	0.489 ***	0.503 ***
(8) Depression score										0.689 ***	0.778 ***
(9) Anxiety score											0.818 ***
(10) Stress score											

* *p* < 0.05, ** *p* < 0.01, *** *p* < 0.001.

**Table 5:** Multiple linear regression analysis with independent variables, including depression, anxiety, and stress scores, and the total PSQI score as the dependent variable (*n* = 386).

	β	t	SE	*p*
Intercept	5.085	23.035	0.221	<0.001
Depression score	0.178	3.987	0.045	<0.001
Anxiety score	0.141	2.386	0.059	<0.001
Stress score	0.081	1.614	0.05	0.107
R^2^	0.29			
Adjusted R^2^	0.285			
F	52.093 *			

SE, standard error; * *p* < 0.001.

## Data Availability

The data assessed and reported here can be obtained from the corresponding author upon reasonable request and following ethical and privacy principles.
